# A Contribution to the Study of Erosion

**Published:** 1893-04

**Authors:** J. Morgan Howe

**Affiliations:** New York


### International Dental Journal.

Vol. XIV. April, 1893. No. 4.

### Original Communications.[1]

### A CONTRIBUTION TO THE STUDY OF EROSION.[2]

BY J. MORGAN HOWE, M.D., NEW YORK.

In presenting the subject of erosion for your consideration this evening, it is more with the hope of eliciting valuable information than adding much myself to the knowledge of the subject. If, however, we make record of all that is observed by us, the aggregate of our facts may soon enable us to have more definite ideas of the etiology of this singular waste of dental tissue.

I think it will be of some service for us to hastily refer to some of the observations of a few of those who have made suggestions or written on this subject.

Twenty years ago Dr. Charles R. E. Koch, of Chicago, in an exhaustive paper, said, "At this time the most general belief undoubtedly is that the disease is a process of chemical dissolution." He also quotes Charles Tomes's citation of an observation of Dr. Murie's that the teeth of a sea-lion had been thus wasted in localities least exposed to attrition.

Dr. L. G. Noel, of Nashville, from the medical point of view, wrote in 1875 that he had observed erosion as one of the results of chronic rheumatism.

---

[1] The editor and publishers are not responsible for the views of authors of papers published in this department, nor for any claim to novelty, or otherwise, that may be made by them. No papers will be received for this department that have appeared in any other journal published in the country.

[2] Read before the New York Odontological Society, January 17, 1893.

In 1879, Dr. R. Finley Hunt expressed the opinion that erosion was caused by an alkaline condition of the oral fluids.

In 1881, in the discussion of a paper read before the American Dental Association, Dr. Litch said that erosion unquestionably required "a vital condition of the tooth-structure for its development," and that he had never seen it occur in a devitalized tooth. And Dr. G. H. Cushing corroborated the statement as to the immunity of such teeth.

In January, 1884, in the discussion of a paper by Dr. E. T. Darby, Dr. Guilford said that when pulpless teeth are found eroded, "it has probably taken place before the teeth lost their vitality." Dr. Darby said that he had one case of erosion in a pulpless tooth, but the next year,—1885,—the same subject being under discussion, Dr. Darby said that he was not sure whether the erosion occurred before devitalization or afterwards. In the latter discussion, Dr. James Truman proposed the theory that acid secretions from the mucous follicles of the lips were the immediate causes of erosion, and that this action took place mostly at night.[1] He suggested that the discovery of the condition was to be made by testing with litmus paper in the morning on rising.

In 1886, Dr. Kirk, in a paper on this subject, said that the idea

---

[1] Dr. Truman's position here is not clearly stated, there being a natural misinterpretation of what he attempted to prove.

The examination of many cases of erosion had failed with him to demonstrate either an acid or an alkaline action. The testing of the oral secretions during the day hours gave no positive response. This led to an examination at night, and immediately on rising in the morning. The results demonstrated a marked acid reaction in every case.

The well-known fact that the salivary glands are inactive during hours of rest led to the conclusion that the process of fermentation proceeded during that period undisturbed by the flow of the alkaline saliva. With such a condition there could be but one result, the production of a decalcifying agent. That this was acid was demonstrated by the tests, and, further, there is no evidence to prove that ordinary alkaline fluids have any effect on enamels.

The action of the lip, according to his hypothesis, was purely mechanical, holding the secretions in close contact to the teeth during the night, and acting as the polishing agent through the day.

Dr. Kirk, confirming Dr. Truman's investigations, carried these further by demonstrating the extreme acidity of the secretion of the mucous follicles of the lip, thus adding another factor in the destruction.

It is at present Dr. Truman's opinion that the fermentation at night, with its resulting acid product, has never received the attention that it merits. It is, in his judgment, important as a pathological factor in all forms of tooth destruction, and cannot be set aside as an element of no value.—Ed.

that alkalies produce erosion " is easily disproved by subjecting teeth out of the mouth to alkaline solutions. He was impressed with the suggestions and experiments of Dr. Truman, and he had repeated and confirmed the latter. His conclusion was that erosion " is caused by the solvent action of the acid secretions," and is in no way dependent on mechanical abrasion.

In 1888, Dr. W. D. Miller reported the result of an experiment in a mouth where erosion was progressing, in which, he said, although we have all seen pulpless teeth presenting erosion, " we have not been able to say that these erosions were not produced while the pulp of the tooth was still alive." His experiment consisted in setting an inlay of ivory in an eroded cavity, with the result showing distinct horizontal furrows with a finely-polished surface in the ivory, about two years later.

In 1890, Dr. Miller recorded the effect of the action of a five- to ten-per-cent. solution of caustic potash, on teeth left in the solution some weeks ; stating that the ends of the roots could be crushed in the fingers, and easily cut with a knife. He remarks that " attempts to account for erosion by the action of decalcifying agents have not thus far led to a satisfactory solution of the question, and it might be well in future, while searching for the cause, to bear in mind that the teeth may be acted upon by agents which attack primarily the organic, as well as by those which attack only the inorganic, constituents."

Professor J. E. Garretson has favored the electro-chemical theory of Mr. Kencely Bridgman, and expresses the opinion that there must be a predisposition, the result of an impression made on the enamel at the formative period.

These few references to the opinions expressed during the last two decades present two salient points for notice. First, the radical differences of opinion expressed, and the contradictory evidence offered, as in Dr. Kirk's easy disproval of the suggestion of destructive action being due to alkaline fluids, and Dr. Miller's experiment seeming to prove to his mind that alkaline solutions were worthy of consideration as active agents.

But the other and more noticeable point in these quotations is the constantly recurring idea that the vitality of the dental tissues was in some way a factor in the destructive process, and a frequent expression of doubt whether pulpless teeth were subject to erosion. I fell into this error myself several years ago, and proposed that the nutrition of the teeth was probably concerned in the disease.

And although Dr. Miller's experiment—in which he obtained eroding action on ivory in the mouth—was relevant, it was hardly to be regarded as conclusive. It was, therefore, with unusual interest that I observed in the mouth of Mrs. N——, in February, 1892, the erosion of a pulpless central incisor, together with similar effects on several other incisors having living pulps.

I had seen this lady's teeth ten months previously, and knew that there was no erosion. There was a history, meantime, of vital depression, with general hyperæsthesia, and in the latter the teeth sympathized. Again, within the last two weeks, I have seen on the teeth of Mrs. H—— the beginning of erosion on the superior left central,—with a vital pulp,—and also on the pulpless right lateral. There has never been any gingival recession or erosion in this lady's mouth, but both of these lesions have affected these two teeth within the past year under my personal observation. There is a history of rheumatism of many years' standing, causing much suffering when she was sixteen to eighteen years old, but since that time it has been less acute and severe, although seldom remaining many months without some painful symptoms. The past year has not been markedly worse than several previous ones in this respect.

The observation made in these two cases that pulpless teeth are subject to erosion came to me as the revelation of a fact that I had not been aware of, and which the records show has been the subject of much doubt with others; but further back I have found it stated, in Maury's Dental Surgery,—1843,—that Dr. E. Parmly had recorded an observation as settling this question "beyond all doubt," that "a gentleman who had lost his teeth, partly from this cause came to us about four years ago . . . to procure artificial substitutes. A set of human teeth of the best quality were accordingly provided, which, in the course of three years and a half, on his return to us, were found to be grooved from the surface to the central cavity;" and this same reference to Dr. Parmly's record is made to-day in Harris's "Principles and Practice." So that this fact,—that pulpless teeth are subject to erosion,—which has been a point of considerable speculation and assumption for years, and which I supposed I had made an original observation upon, was quite fully settled by Dr. Parmly's observation of fifty years ago. We may positively exclude, then, all further consideration of the vitality of the eroded tissue, or of its nutrition, as being a factor in its destruction. And we are able to confine our attention to the quality of the fluids of the mouth, which must be—it would appear

—the immediate cause of the solution of the tissue. That is to say, we may hope to find in the oral fluids an explanation of their solvent action, but the etiology of erosion, of course, is to be sought for in general systemic conditions.

In the case of Mrs. N——, before referred to, I tested the reaction of the labial mucous membrane with blue litmus, and furnished her with paper to make tests, in the morning on rising, and at other times. The result was that a decidedly acid reaction was almost always shown, and this was somewhat more pronounced in the morning than at other times. This was, I think, the first time I had obtained a decidedly acid reaction in such cases, although I had made tests many times; but, while considering the case, I placed a piece of blue paper under my own lip, with the result that when it was moistened with the mucus it showed as distinct acidity as the reaction from the mouth of Mrs. N——. I repeated this test in my own mouth many times, on different days, and with varying intervals, always getting some sign of acidity and often very decided change of the blue to pink, and there had never been any erosion in my mouth. This being in February last, I waited for results in my own case, thinking that perhaps erosion would appear on my teeth.

Upon the appearance of the new case of erosion on the teeth of Mrs. H——, before referred to, I tested the mucus of her labial mucous membrane, as in the former case, and was not able to get quite so distinct acid reaction at any time, as in the former case, and most of the tests made in her mouth, even in the morning on rising, showed little or no acidity. While obtaining these latter tests I again made a series of tests of the mucus from my own lip, and found it quite acid on many different days. The tests in the morning on rising seldom showed any acidity; but in the forenoon, between ten and twelve o'clock, there was almost always decided acid reaction. My general health has been good during the past year, and there is as yet no erosion of my teeth. I give these facts for what they may be worth, but they appear to suggest much doubt as to the significance of mere acidity of mucus; at least it is shown that the latter quality may exist without erosion occurring very speedily.

An interesting and important question is raised in connection with the consideration of this subject,—namely, how can we tell when the process of erosion has stopped? I have seen cases of erosion in which it seemed to me that for several years there was no destructive progress made, but it was never so certain that I

could positively claim that the disease was arrested. If one or more eroded cavities are filled, the filling-material often furnishes a means of measuring the rate of destructive progress, or the fact of its arrest; but without such means of mechanical measurement, I think the determination doubtful. Eroded teeth that are sensitive to irritating agents have seemed to me to waste sometimes more slowly than some others that were very little, if at all, sensitive. Is sensitiveness of the wasted surface a guide to the determination of the progress of the disease?

In May last, Dr. H. C. Meriam said, in a meeting of the American Academy of Dental Science, that he had two years previously "completely cured a case of erosion by the use of a wash." I have written Dr. Meriam about this case, and a portion of his letter in reply is presented to add to the interest of the discussion of this subject. But, valuable as his suggestions and his testimony are, there arises the question, How does he *know* when erosion is stopped?

*Dr. H. C. Meriam.*—What is the state of the blood of the patient? In some cases I have seen the patient was rheumatic. In rheumatism the blood is acid, so much so that bad cases smell sour; uric acid is found on the skin. "Through the skin are exhaled the watery parts of the blood." "The mucous membranes have a striking analogy with the cutaneous tissues in organization and diseases." The above from Dunglison's Medical Dictionary. It would be interesting to know if the watery parts of the blood are acid in diseases of the skin. A dermatologist might give us some light on this.

When the case reported at the meeting of the American Academy of Dental Science, in May last, came to me, I was struck by the appearance of the surface of the teeth, resembling that of some large pearl shells that I cleaned with acid when a boy; and also a bad case of erosion that I had seen due to excessive use of lemons. I had never accepted Dr. Tucker's theory of the action of tannin on the teeth, but believed the benefit derived, if any, to be due to its action on the glands of the mouth.

The surfaces of my patient's teeth looked like cathedral glass, and it seemed to me that they were being acted on by acid over their entire surface; no labial or buccal cavities were forming, the action being limited, as far as I could see, to the upper front teeth.

What should I do? Direct an alkali which would clear the mouth for a time, or attempt to change the character of the excretions by treating the mucous glands. I therefore directed my patient to use alcohol three times a day, using enough to get a burning sensation, thus giving tone to the membrane, and getting a glow, just as we do after a cold bath. It would seem we should extend our inquiries to the general system and try to act with the family physician if general conditions are noticed. I do not wish to commit myself to alcohol or anything as a specific, but I have one grateful patient, and shall continue to use it, holding myself ready to change as truth advances.

I am disposed to think the glow that follows its use to be the valuable part of the treatment, and perhaps we may find an agent of more value than alcohol. The condition of the blood in fevers, rheumatism, and uterine disturbances should, I think, be looked into, and a physician, who had made a special study of rheumatism, would, I think, be able to help us.

